# Sexual Harassments Related to Alcohol and Drugs Intake: The Experience of the Rape Centre of Turin

**DOI:** 10.3390/ijerph192215090

**Published:** 2022-11-16

**Authors:** Barbara Mognetti, Marco Bo, Giovanni Nicolao Berta, Antonella Canavese, Paola Castagna, Federica Collini, Veronica Santa, Alberto Salomone, Sarah Gino

**Affiliations:** 1 Department of Life Sciences and Systems Biology, University of Turin, via Accademia Albertina 13, 10123 Turin, Italy; 2 Hospital Medical Direction, Local Health Trust TO5, Piazza Silvio Pellico 1, 10023 Chieri, Italy; 3 Department of Clinical and Biological Sciences, University of Turin, Regione Gonzole 10, 10043 Orbassano, Italy; 4 Centro Soccorso Violenza Sessuale, Sant’Anna Hospital, City of Health and Science, Corso Spezia 60, 10126 Turin, Italy; 5 Department of Surgical Sciences, University of Turin, Corso Dogliotti 14, 10126 Turin, Italy; 6 Department of Health Sciences, University of Eastern Piedmont, via Solaroli 17, 28100 Novara, Italy; 7 Corso di Laurea Magistrale in Medicina e Chirurgia, Azienda Ospedaliera Universitaria San Luigi (Orbassano), University of Turin, v. Verdi 8, 10124 Turin, Italy; 8 Department of Chemistry, University of Turin, via Giuria 5, 10126 Turin, Italy; 9 Centro Regionale Antidoping, Regione Gonzole 10/1, 10043 Orbassano, Italy

**Keywords:** gender violence, sexual violence, rape, drug intake, alcohol intake, DFSA, injuries

## Abstract

A cross-sectional study was conducted that describes the characteristics of sexual violence episodes related to the intake of alcohol and drugs observed among women that turned to the “Centro Soccorso Violenza Sessuale” (SVS) of the Sant’Anna Hospital in Turin between 1 January 2008, and 31 December 2017. Two hundred twenty-two patients were enrolled, 25 of which were minors, 141 were Italians, and most of them knew their aggressor and were raped in a private home. One hundred and fifty-five of them declared to the healthcare personnel to have taken alcoholic substances and/or drugs in conjunction with the event (86 reported having drunk alcohol, 36 having taken drugs and 33 disclosed both alcohol and drug abuse). If the woman knew her abuser, alcohol consumption was described as voluntary in more than 80% of cases, while in relation to drugs the consumption was equally voluntary or fraudulent. About 73% of women who reported having drunk alcohol just had amnesia or amnesia related to other symptoms, while amnesia was present in about 63% of women who reported only drug use. Physicians observed physical injuries on 156 women. Patients who reported to have assumed alcohol presented a significantly higher risk to suffer any physical injury and have a significantly increased risk to suffer injuries to their head and/or neck. The results obtained underline how even in Northern Italy alcohol intake represents the most widespread psychoactive substance in case of drug-facilitated sexual assault. There is therefore a need to promote education and prevention campaigns among citizens, especially among the youngest.

## 1. Introduction

All sexual acts unwanted and often obtained by violence or coercion or through psychological and/or physical threats are considered sexual harassment. Any form of unwanted intimacy represents a violation of the dignity and freedom of the individual. For this reason, it must be deemed as a violation of human rights. However, this form of violence is still underestimated, not recognized, or more often silenced, because of socio-cultural aspects or linked to the victim’s fear of the consequences of a possible complaint. Sexual violence represents a transversal phenomenon, which mainly affects women of every social, ethnic, and religious group, regardless of the victim’s age.

The data deriving from international surveys [1,2] confirm the importance of programs aimed at stopping this phenomenon by means of legislative [3], communication, education and health-related tools including training of health personnel and drafting of guidelines [4,5].

A close relationship between alcohol intake and being a victim of violence has been demonstrated: its consumption can facilitate forms of physical abuse, but also represents a form of addiction resulting from the violence suffered by the victims [1,2]. A survey conducted in 2014 by Larsen and colleagues [6] confirmed the close correlation between violence against women and alcohol use, highlighting frequent consumption [7,8] by the victims shortly before the event, especially for the younger ones. The disinhibiting action of alcohol can induce behaviours that are not normally proper to the person and at the same time can decrease the victim’s perceptive and defence reaction skills [9]. Additionally, it is not so unusual that those victims declare the intake of prescription drugs [1,2,10,11] that affect the activity of the central nervous system: the effects of alcohol itself can therefore be enhanced by the simultaneous intake of these molecules.

Besides alcohol, over the past twenty years, the literature has also reported the correlation between sexual violence and psychotropic substances [1,2,7,8,10,11], namely benzodiazepines, benzodiazepine analogues or “Z drugs”, and drugs of abuse [7,8,12,13,14,15]. As well as alcohol, substances such as cannabinoids, first-generation antihistamines, amphetamines and γ-hydroxybutyric acid (GHB) are reported in the literature as rape-facilitating [10,16,17], because they can alter consciousness, perception and reaction capacity and awareness when it comes to consent [18]. The “rape drugs”, whose main consumption occurs in recreational situations, are used in cases of aggression because of their short half-life and their difficult identification in the toxicological investigation, without forgetting their amnesic effect on the victim. Beside the most famous flunitrazepam (better known as Rohypnol^®^) are listed 3,4-Methylenedioxymethamphetamine (MDMA, or ecstasy), GHB, ketamine, amphetamines, and lysergide (LSD) [7,8,10,11]. The victim can take them voluntarily, in a forced way or by a fraudulent administration. In the latter case, the administration generally occurs by unknown subjects, especially in places of aggregation, without underestimating the possibility that it can be carried out by a partner or by an acquaintance.

Since the 1980s, forensic toxicology has seen the evolution of a flourishing scientific literature aimed at demonstrating in some cases the possible link between violence and the use of substances by the victim. The literature identifies a specific typology definition of this type of crime, namely drug-facilitated sexual assault (DFSA) [7,8,9], be it proactive (a violence perpetrated following a fraudulent or imposed administration of a substance, aimed at making the victim vulnerable and uninhibited) or opportunistic (an act of a sexual nature perpetrated against an unwilling victim, previously made vulnerable by the voluntary intake of substances).

Despite DFSA being underestimated and probably much more frequent than reported [19], there is a lack of literature studies considering all the different aspects of the aggression (the type of substances ingested, their mode of administration, the psychological and physical symptoms at the time of the visit and many more). To fulfil this gap, we conducted a cross-sectional study to describe the characteristics of sexual violence episodes related to the intake of alcohol and drugs observed among women that turned to the “Centro Soccorso Violenza Sessuale” (SVS) of the Sant’Anna Hospital in Turin. As secondary objectives, the study aims to evaluate which factors are associated with a positive result for alcohol or drugs at toxicological investigation and whether there is a relation between what women declared or what healthcare suspected at the victim’s arrival to the SVS regarding alcohol and/or drug use and the results of the toxicological investigations.

## 2. Materials and Methods

### 2.1. Study Design

We conducted a retrospective cross-sectional study, based on data collected in the medical records of sexually abused female patients, who were examined at the Rape Centre “Centro Soccorso Violenza Sessuale” (SVS) at Sant’Anna Hospital in Turin, Italy, between 1 January 2008, and 31 December 2017. The SVS is one of the two Italian rape centres open 24 h per day, 7 days a week. It is the reference centre for the metropolitan area of Turin in North-West Italy, which counts about 2,200,000 inhabitants. With its multidisciplinary team (gynaecologists, midwives, psychologists, social workers, and medical examiners), the SVS takes care of sexually abused women, mistreated pregnant women and migrant women who have suffered any form of violence.

We included in the study all patients aged 14 years or older, who reported sexual abuse and declared alcohol and/or drug intake or had physical and/or psychological signs and/or symptoms that guided SVS healthcare professionals to suspect alcohol/drug use, and for whom a complete medical record and mass spectrometry-based toxicology test results were available.

The research protocol was organised according to The Code of Ethics of the World Medical Association (Declaration of Helsinki) for experiments involving humans (2013), to the General Data Protection Regulation (2018) and to Provision no. 146/2019 of the Italian Privacy Guarantor. The study was approved by the Ethics Committee of the “A.O.U. Città della Salute e della Scienza di Torino—A.O. Ordine Mauriziano di Torino” (CE 112/2020) on 6 July 2020.

### 2.2. Data Collection

For issues related to the protection of privacy, the medical records are papery and kept in locked files in dedicated places accessible only by the staff of the Centre. The data was extracted from one author and checked by a second author who is on the staff of the SVS Centre. We collected relevant information about violence episodes from the women’s health records and then registered them into a database administered by the SVS staff (see Supplementary Item S1). We looked for socio-demographic data (age and nationality); the women were classified into 6 age groups (14–17; 18–19; 20–29; 30–39; 40–49 and over 50 years of age). Considering nationality, patients enrolled were categorised as “Italian” and “foreign”. We noted the characteristics of the episode of violence: where it took place, the relationship with the aggressor, the number of attackers and the time elapsed from the violence to the medical examination. Data on the drugs and substances that the women declared to assume, occasionally or as prescriptions, were also collected. Signs and symptoms, the type of injuries and the body area involved were used to classify the episode.

We also evaluated psychological conditions and psychic symptoms: according to the guidelines in force in our region, when a woman who is a victim of abuse accesses the SVS, a series of symptoms, itemized in a checklist contained in the violence kit, is evaluated and registered on the medical record by specialized medical staff (psychologist or psychiatrist as needed). Among the recorded symptoms, we decided to consider only the conditions most frequently associated with the intake of drugs or alcohol (i.e., anterograde amnesia, hallucinations, numbness, confusion). In the analysis phase, the recorded data were encoded as described in Supplementary Item S2.

Lastly, we investigated on reports sent to the Judicial Authority by healthcare professionals and on the complaints done by women before or at the time of the medical examination. On the basis of the data collected we organized categories for the different variables analyzed in order to make everything exploitable for statistical evaluation.

### 2.3. Toxicological Findings

We considered both prescriptions and any drugs administered at the emergency room. We also carefully evaluated patients’ stories to identify any voluptuous substances (alcohol and drugs) both being declared as taken voluntarily, forcibly or unconsciously in conjunction with the episode of violence.

The results of the toxicological analyses were examined in relation to the time elapsed between rape and medical examination at the SVS, the age of the women, the relationship between victims and assailants, the injuries described, the type of biological samples collected (blood, urine, and hair).

The substances have been divided into several categories based on their ATC classification: anaesthetics, cannabinoids, opioids, sedatives, antipsychotics, antidepressants, other drugs that affect the central nervous system (CNS), other drugs that do not affect the CNS, and alcohol.

### 2.4. Statistical Analysis

Descriptive data are shown as absolute and relative (%) frequencies of the different modalities for categorical variables.

We performed univariate analysis—using chi-square test or Fisher’s exact test, as appropriate—to verify the association between having suffered any physical injury and having suffered injuries to head and/or neck, between knowing the aggressor (or aggressors) and having suffered injuries to head and/or neck, between the places where the rape happened and the type of consumed substance and the modality of hiring, between having assumed alcohol or drugs and the time elapsed from rape and medical examination, between having assumed alcohol and having suffered any physical injuries and having suffered injuries to the head and/or neck and between a toxicological analysis positive for alcohol and nationality, age classes (14–19 years, 20–39 years and ≥40 years), knowing the aggressor, having declared assumption of alcohol (alone or in combination with drugs), having declared use of drugs, having received medical examination till 48 h from rape, amnesia and having suffered any physical injury. For the categorical variables, we also calculated odds ratios (OR) and their confidence intervals (IC95%).

To adjust for the relative effect of each factor associated with a toxicological examination positive for alcohol or for drugs, we performed a multivariate analysis with the variables that were found to be significantly associated with the probability to obtain a positive result at toxicological examinations in the univariate analyses. Specifically, we designed a logistic regression model for the positive toxicological examination of alcohol and drugs, respectively. Then, we calculated Spearman’s correlation coefficients between a declared alcohol intake (alone or with any drug) and a toxicological analysis positive for alcohol and between a declared use of any kind of drug (alone or with alcohol) and a toxicological analysis positive for the drug. We also stratified both analyses by nationality, time from rape to medical examination, having suffered amnesia and kind of sample used for toxicological analysis.

Statistical significance was set at *p* < 0.05. All data were analysed with Stata 13 (StataCorp LP, College Station, TX, USA).

## 3. Results

### 3.1. Population Enrolled

We evaluated the medical records from 1204 patients, who were examined over the study period. Two hundred twenty-two patients met the inclusion criteria, 25 (11.3%) of which were minors. One hundred forty-one women (63.5%) were Italians and 81 (36.5%) were foreign. Of the latter, 51 (63.0%) women came from non-European countries. Table 1 describes the demographics of the enrolled patients in detail.

**Figure 1:**
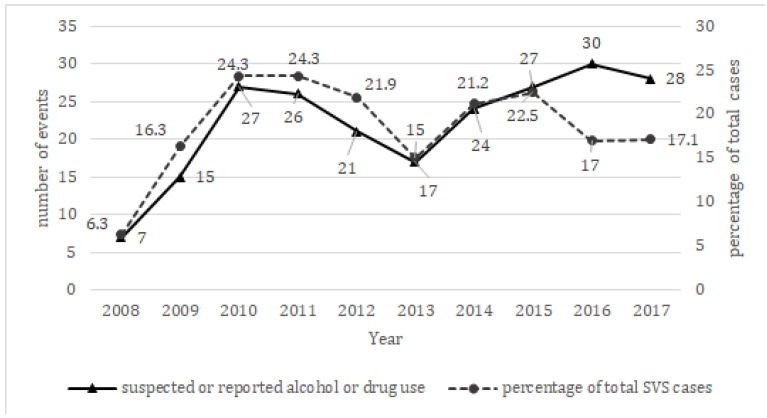
Suspected or reported alcohol or drug use: numbers of sexual violence events related to a suspect or reported alcohol and/or drugs intake detected by year (black line, right *Y* axis) and their relative percentage towards total number of cases accessing the SVS (dotted line, left *Y* axis).

The number of events in which health workers took notice of a suspected or reported alcohol and/or drug intake at the medical examination is shown in Figure 1. Over time there has been a bimodal distribution in the cases detected by the staff of the SVS Centre; the absolute numbers and proportions of events, in which health workers took notice of a suspected or reported alcohol and/or drug intake at the medical examination showed a four-time increase between 2008 and 2010–2011 when women with suspected or reported alcohol and/or drug intake reached a quarter of the examined ones. From 2013, the number of cases with suspected or reported alcohol and/or drug intake proportionally increased with the increase in the number of women who turned to the rape centre, representing 17% to 24% of the examined events.

**Figure 2:**
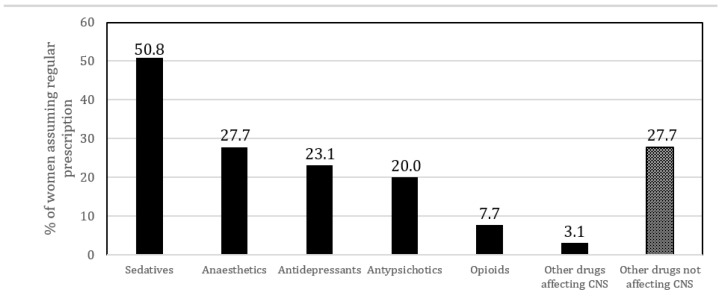
Usual prescriptions reported by 65 patients.

From the medical records, it emerged that 65 women (29.3%) reported that at the time of the event they were taking prescriptions on a regular basis assuming one or more medications and that 33 (50.8%) of them were taking sedatives (Figure 2). Only 11 out of the 65 women assuming prescriptions were taking exclusively medications not affecting CNS.

Furthermore, at the Emergency Room medications were administered to 25 women: four patients received anaesthetics, thirteen sedatives, two other drugs affecting the CNS, and six other drugs not affecting the CNS.

### 3.2. Characteristics of Alcohol/Drug Intake, Time Elapsed from Rape and Medical Examination, Features of the Event

Of the 222 women enrolled, 155 (69.8%) patients declared to the healthcare personnel to have taken alcoholic substances and/or drugs in conjunction with the event. In particular, 86 (55.5%) of these women reported having drunk alcohol, 36 (23.2%) having taken drugs and 33 (21.3%) disclosed both alcohol and drug abuse. The intake of alcohol and drugs was voluntary in, respectively, 87.4% and 43.5% of the cases, while it was described as forced or supposed to be unconscious by 11.8% and 55.1% of the women. Four women declared no intake, while for the remaining 63 no data on alcohol/drug intake were available on the medical records.

One hundred twenty-three out of 222 women included (55.4%) received a medical examination at the SVS within 24 h from the rape; the time elapsed between the rape and the medical examination is unknown in 8 cases (5.2%) (Table 2).

Most of the women (76.5%) who declared alcohol consumption (alone or in combination with drugs) went to the hospital within 48 h (rising to 81.4% in those who consumed alcohol alone), while most of the women who reported only drug use (38.9%) received medical examination after 48 h from rape (Table 2). Coherently, having consumed alcohol (alone or in combination with drugs) is associated with a significantly higher probability to undergo medical examination within 48 h from rape (OR: 2.69; IC95%: 1.14–6.35).

Table 2 also compares the age of the woman and the type of substance taken: most of the women aged 18–39 declare they had drunk or taken both alcohol and drugs in conjunction with the event.

Most of the enrolled patients knew their aggressor, being the partner in 22.5% or another known man in 33.8% of cases. Forty-eight (21.6%) women reported having suffered sexual violence by more than one assailant and about 35 (72.9%) of them did not know their aggressors. No information about the aggressor was found in 23 medical records (Table 2). If the woman knew her abuser or her assailant, alcohol consumption was described as voluntary in more than 80% of cases, while in relation to drugs the consumption reported in medical reports was equally voluntary or forced or supposedly unconscious.

Although the place where sexual violence was carried out was not recorded for 9 women, 141 of them (63.5%) were raped at a private home, 42 (18.9%) in a public place, 5 (2.2%) at the workplace, while 25 (11.3%) women could not refer where they were raped. No significant differences were highlighted between the places where the rape happened and the type of substance that women referred to having used and how substances were taken (voluntary, forced, supposed unconscious).

### 3.3. Clinical Presentation

The clinical picture of these women was characterised by some recurrent physical symptoms, such as widespread pain and nausea, but only 27 (12.2%) women reported more than one physical symptom. Regarding psychic manifestations, about half of the women in our sample had multiple symptoms such as anger, sadness, fear, numbness, and confusion frequently associated with amnesia. About 73% of women who reported having drunk alcohol just had amnesia or amnesia related to other symptoms, while amnesia was present in about 63% of women who reported only drug use.

During the medical examination, physicians observed external lesions on 156 (70.3%) women (Table 2), but only 117 (52.7%) medical records described them in detail. Lesions are mainly represented by abrasions and bruises. Of the women with injuries, approximately 97.4% had at least one blunt force injury and 4.4% had at least one blade injury. Supplementary Table S1 summarises the type of lesions recorded. Regarding the regions of the body affected by injuries, 48 out of the 156 women reporting lesions (30.7%) had injuries exclusively at the head and the neck, 14 (9.0%) at the genital region and/or breasts (erogenous zones), 43 (27.6%) at multiple not erogenous zones, and 25 (16.0%) at multiple sites, including genitals and/or breasts. The remaining victims of sexual violence had injuries limited to the trunk (6), upper limbs (10) or lower limbs (10). The majority (95%) of women with head and neck injuries reported having taken only alcohol or alcohol plus drugs, as did 69.2% of patients who reported genital/breast injury, regardless of whether this was the only region of the body offended.

Looking at women who took alcohol alone or in combination with some drugs and patients who reported only drug intake, there is a statistically significant difference in the occurrence of injuries. In particular, patients who reported to have assumed alcohol presented a significantly higher risk to suffer any physical injury (OR 19.0; IC95%: 1.19–303.99) and have a significantly increased risk to suffer injuries to their head and/or neck (OR: 2.25; IC95%: 1.06–4.76).

Considering the different age groups, most minors and women over 50 had no injuries, while head and neck lesions were more frequently found in women aged 18 and 19 years (Table 3).

Table 4 describes the data relating to the site of the injuries with respect to the category of the aggressor: the patients who declared to know their aggressor (or aggressors) have about a five times higher risk to suffer injuries to the head and neck (OR: 4.72; IC95%: 1.70–13.10).

### 3.4. Reporting to the Judicial Authorities

In 183 cases (82.4%) the medical staff of the SVS reported the rape to the judicial authority, while we found no information regarding a medical report in the medical records of 13 (5.9%) women. As for the willingness of women victims of violence to file a complaint, out of 222 women only 47 (21.2%) declared that they had already done so.

### 3.5. Toxicological Findings

Specimens for toxicological analysis were collected in all cases examined in the study: both blood and urine samples were taken from 126 (56.8%) women while, for 16 (7.2%) women, hair was the only biological sample collected by the SVS healthcare personnel. More details about the sampling are reported in the Supplementary Materials.

Samples were positive for alcohol and/or substances in 141 (63.5%) sexually abused women. Twenty-one (9.5%) patients were positive only for alcohol, while the association of drugs (including medicaments) and alcohol was found in 19 (8.6%) cases (Table 5).

Out of the 81 negative toxicological analyses, 30 (37.0%) regarded women examined more than 24 h after the event. In 10 out of 16 cases, in which the only biological material collected was represented by hairs, the toxicological analysis showed the intake of sedatives, opioids and anaesthetics.

The substances most frequently detected, whatever the biological matrix collected was, are represented by sedatives, alcohol and drugs that do not act on the central nervous system (Table 6).

In Supplementary Table S2 toxicological findings are stratified by the time elapsed from rape to medical examination, the age class, the characteristics of the perpetrator and the injuries detected at medical examination.

A toxicological analysis positive for alcohol was significantly associated with being Italian (OR: 3.8; IC95% 1.4–10.3), having declared the assumption of alcohol (OR: 20.5; IC95% 2.2–187.4), having received medical examination till 48 h from rape (OR: 9.0; IC95% 1.9–43.1), amnesia (OR: 2.7; IC95% 1.0–7.3) and having suffered any physical lesion (OR: 4.8; IC95% 1.3–17.4). We found no statistically significant association between a positive toxicological analysis for alcohol and age classes, nor knowing the aggressor. The probability to obtain a positive result at toxicological analysis for alcohol significantly increased only with having declared the assumption of alcohol and having received a medical examination till 48 h from rape at multivariable analysis (Table 7).

We found a statistically significant correlation between having declared to have consumed alcohol (with or without the use of drugs) and a toxicological analysis positive for alcohol (Rho = 0.37; *p* < 0.01). We also found that such correlation is slightly higher among stranger women (Rho = 0.59; *p* = 0.02), among those who received medical examination till 24 h from rape (Rho = 0.48; *p* < 0.01), among those who did not suffer amnesia (Rho = 0.44; *p* = 0.02) and when toxicological analysis was performed on blood samples (Rho = 0.42; *p* = 0.02).

We found no statistically significant correlation between having declared to have consumed drugs (with or without the use of alcohol) and a toxicological analysis positive for any drug.

## 4. Discussion

The findings of a recent review suggest that “a diverse range of substances are associated with DFSA” and that consumption of recreational substances at social events renders victims more vulnerable. As such, typical DFSA cases are opportunistic in nature, and mainly involve women in their mid-twenties and an acquaintance as the perpetrator [20]. Our study considers data regarding women 14 years old and over, victims of sexual violence, examined at the main Rape Centre of the Italian Piedmont region in ten years (from 2008 to 2017) that reported sexual abuse, with a declared alcohol and/or drug intake or where the health personnel suspected it based on the clinical presentation. About two third of the women were Italians, different from what Bertol described in a study conducted between 2010 and 2018 in Florence (Hospital Careggi) [16]; more than half of the victims were between the ages of 18 and 29, the mean age being slightly lower than what described in the above-cited analysis but in line with data highlighted by the Italian Institute of Statistics (ISTAT) in a follow-up survey on violence against women in Italy [19].

The number of cases included in our study gradually increased during the first two years, while in the last four years (2014–2017) there was an almost constant trend with an average of about 27 cases/year. From 2011 onward such an increase was proportional to the increase in the number of women who turned to the Rape Centre. The rise observed in the first two years could be explained in two different ways: on the one hand, there could have been, a real increase in alcohol and drugs self-taken or administered to make the victims incapable or uninhibited; on the other hand, it could be due to a greater awareness of survivors determined by prevention/information campaigns and a lower sense of shame to report voluntary use of illicit substances before the event, but also to the greater training and awareness of health personnel on the management of the victims of gender-based violence.

Moreover, such attention must be considered especially within the relationship between the victim and the perpetrator, because in more than half of the cases the aggressor was the partner or a known person and the violence occurred in most cases in a private home, as frequently described in the literature [1,2,21,22,23,24].

Our study does not discuss only the use of substances declared or presumed at the time of the medical examination with the results of toxicological tests, but it appears to be among the few studies that correlate the intake of substances of abuse with injuries found at the time of the visit. Unlike other studies [25,26] that describe in depth only anogenital lesions, while physical signs in other body areas are classified as serious, moderate, or minor, our work classifies injuries according to the localization, the number of injured sites (single or multiple), the type of injury (contusion, stabbing or firearm), and the substance intake (alcohol or drugs). In our sample, physicians observed external lesions on 70.3% of women; this value is higher than what was described in sexual violence events in Norway by Hagemann (66.5%) in 2011 [25] and in two different studies published in 2021 related to Lombardy region (North-Centre Italy) by Maghin (61%) [22] and by Torazzi (respectively 44.9% in adolescent girls and 52.1 in adult women) [24], but less than was observed in Denmark by Ingemann-Hansen (78%) [23].

Although women who reported drinking alcohol have a higher risk of being injured, generally in our study taking alcohol or drugs increases the risk of trauma (especially blunt wound); the regions most involved are the head and the neck, especially in younger patients (aged between 18 and 29 years) and predominantly abused by their partner or a known person. Much is known about the head and neck injuries associated with the experience of domestic violence since the early 2000s [27,28,29,30]. Our results are also consistent with some more recent studies. A high frequency (nearly 71%) of head and neck wounds in women victims of family and domestic violence has been reported also in an Australian retrospective study [31]: about half of them (47.9%) were injured in the head and neck regions. A similar result was reported by Ucar et al. [32] whose study shows that nearly half of the women who presented to the Emergency Department due to Intimate Partner Violence (47.9%) had trauma at the head and neck regions. In this case, the authors highlighted that women presenting to the emergency department for evaluation and management of orofacial injuries (unless related to motor vehicle accidents) should be considered at high risk for domestic violence [30]. Based on such evidence, the hypothesis has been advanced that head, neck, and face injuries may be clinical markers of Intimate Partner Violence.

Moreover, in our study 20 women wounded at the upper (10) and lower (10) limbs were documented, possibly indicative of self-defense and therefore of the women’s ability to react. As described by Tiemensma [21] the most common physical and psychic symptoms declared by our survivors are nausea, confusion, and partial and total amnesia (Supplementary Materials Table S3). Coherently, even in our study 73% of women who drank alcohol and 63% of patients that took drugs reported amnesia.

In our sample the consumption of alcohol and drugs declared at the time of the medical examination is almost comparable to that of drugs alone, unlike what was reported by Maghin [22]; on the other hand, the consumption of alcohol alone appears to be two and a half times that of the other two conditions. Alcohol intake associated with sexual violence has been described by several authors [7,33,34,35], especially among younger victims (16–24 years) [36]. In our sample, cases with declared consumption of alcohol prevailed in the group of women aged 20 to 29 years, while the ones who denied any substance intake are mostly the youngest ones and those older than 50 years.

From the anamnestic reports, it appears that in most cases the intake of alcohol was voluntary, while forced or supposedly unconscious intake is significantly more frequent for drugs. These results are consistent with available literature that asserts that voluntary consumptions present the greatest risk factor for DFSA [9,12]. They also suggest and highlight once again the importance of prevention in spreading information, especially to the youngest age groups and in their awareness concerning the risk of assuming alcohol and drugs.

In this Italian sample, the toxicological analysis most frequently highlighted the consumption of sedatives, alcohol, drugs not active on the CNS and cannabinoids. About one-third of the victims took sedatives, anaesthetics, antipsychotics, and antidepressants for therapeutic use. These results are in line with the available evidence, showing that some usual drug therapies may play an important role during sexual assaults or rape. In fact, even if they are prescribed medications, their psychoactive mechanism makes them “facilitating drugs”, because they alter the will or the conscience and can interact with alcohol or other CNS-affecting substances [37].

The results of the toxicological analysis performed sound coherent with “the clinical history” reported by women. In fact, in 63.5% of the cases, there were positive laboratory results, considering that 70.3% of women declared voluntary intake of alcohol and/or drugs at admission at SVS; our results are consistent with what was described by Hagemann (59%) [13] and Tiemensma (67%) [21], but less than what Fiorentin (78.4%) highlighted [38]. Indeed, the probability to obtain a positive alcohol test was significantly higher among women who declared to have drunk alcohol and we observed a significant correlation between having declared alcohol intake (with or without the use of drugs) and a positive alcohol test.

Our study also remarks that the timing and the way of sampling are fundamental to increasing the reliability of the toxicological analysis. Indeed, in a sample where most of the women who assumed alcohol promptly went to the SVS, we observed a stronger positive correlation between alcohol consumption and a positive alcohol test when women received a medical examination within the first 24 h from rape (62% in our sample) and having received medical examination till 48 h from rape also significantly increased the probability to obtain a positive alcohol test.

The possibility of obtaining useful information for forensic purposes from laboratory analyses is however also closely related to three fundamental parameters: the timing of sampling, the adequate training of the operator with respect to the choice of the type of sample, and the execution sampling itself [4].

The biological matrices used in this type of context for toxicological investigations are blood, urine, and keratin (hair or, where not available, hair formation): the choice of the matrix is consequent to the history of the victim, but above all to the time spent between the event and the access to health services [39].

Further, the SVS medical staff collected both blood and urine samples for toxicological analysis from more than half of the examined women, a fact that influenced the correlation between alcohol consumption and a positive alcohol test, which was stronger when the toxicological analysis was performed on blood samples. On the contrary, we found no significant correlation between having consumed drugs and having obtained a positive result in toxicological analysis, a result that may have been influenced by the higher time elapsed between the intake and medical examination in these survivors. In this scenario, it is fundamental not to forget the forensic role of the health personnel in taking not only the toxicological samples but also in sampling biological material for forensic genetics analysis [40,41,42] and in documenting all body injuries [4,22]. Healthcare personnel must therefore be aware of the time windows [7,21,38,43,44,45] within which the tests can guarantee a correct degree of reliability: the blood matrix can be evaluated within 48 h of the presumed event, while the urine can guarantee reliable results up to 5 days later. The choice of the keratin matrix is to be evaluated for cases in which the elapsed time is greater than the times indicated above or if it is necessary to investigate a longer period [38,39,40,41,42], due to the growth time of the hair bulb.

About the disclosure to the judicial authority, the study underlines how the health staff is informed and prepared on the need to file ex officio denunciation in cases of sexual violence in which the assumption of substances is declared or suspected, substances that may affect the ability to understand and the will of the victim, as emerges also in Maghin’s study for sexual violence against adult women [22]. On the other hand, in line with what was reported in 2015 by ISTAT, women refer to the Judicial Authority with reticence: only 20% of our sample filed a complaint and violence is still seen as something to be ashamed of [19].

Our study has its limits. First, the sample consists mainly of adult Italian women with limited representations of other ethnic groups. This fact may have limited the power of the study to describe the role that social and cultural factors play on both alcohol and drug use in rape victims and some of the other characteristics of rape episodes. However, this finding is in contrast with what some of us described in relation to abused pregnant women who turned to the same rape Centre. In that case, approximately 70% of the patients were foreigners [46]. The period of time analyzed stops at 2017, and this undoubtedly represents a limit of our work, but the period of time analyzed still seems significant to us. Furthermore, despite the long period analysed (10 years), the number of subjects enrolled per year is limited and could have negatively influenced the possibility of evaluating the role of some factors that can influence the results of the toxicological analysis, in particular for drugs. In fact, the limited proportion of survivors who reported having intake drugs (less than a third) and the variability of the licit and illicit drugs that women may have taken have probably influenced the ability to effectively describe the factors that can influence the relationship between drug use and toxicological test results and highlight the relationship between drug use and laboratory test results. In DFSA cases, forensic laboratory investigations play a fundamental role in the process, due to the complexity of the investigation based on specific guidelines [7,39,47,48,49] and dedicated resources. The rapid evolution and commercialization of new substances of abuse represent the greatest challenge in the forensic field [16], thus requiring an enormous effort, especially from the laboratory point of view, for a constant updating and development of new techniques that can guarantee a greater investigative capacity and reliability of results [48,50,51]. Some previous studies have stressed the importance of the efforts concerning not only prevention [27], but also metabolization of such new drugs and their effects on behaviour and willingness [50], remembering that also therapeutic drugs and medications can interact and play a significant role [51]. For these reasons, from a medico-legal point of view, it is often difficult to demonstrate a relationship between a woman’s testimony, clinical aspects, and laboratory results, thus limiting the probative value in the forensic context [40,52,53].

To better describe this relationship in our country, it would be useful to conduct further studies involving more health facilities to have a greater number of subjects enrolled.

## 5. Conclusions

The episodes of sexual violence related to the use of substances represent only the summit of an underground phenomenon, whose correct estimation is difficult and constitute an important challenge for operators dealing with women’s health. Many aspects contribute to this bias, among which the victim’s difficulty in remembering events (more than half of the raped women declaring alcohol/drug intake reported amnesia), the fear of not being believed, the delay in accessing services, the use of rapidly metabolized or non- identifiable substances absent from the targeted survey panels.

The results obtained underline how even in our country alcohol represents the most widespread psychoactive substance in the case of DFSA, namely declared consumption of alcohol prevailed among raped women aged 20 to 29 years. Moreover, they confirm that in most cases the aggressor is an acquaintance of the victims and that taking alcohol/ drugs increases the risk of extra genital injuries in raped women. There is therefore a need to promote education and prevention campaigns against gender-based violence among citizens, especially among the youngest, namely considering that few women filed a complaint: violence is still something to be ashamed of. Furthermore, the study photographs the reality of the DFSA perpetrated in a period prior to the SARS-CoV pandemic, it could be interesting to understand if and how this type of abuse may have changed during the lockdown imposed by the pandemic.

## Figures and Tables

**Table 1:** Demographics of the population enrolled.

Demographics	Number (% of the Total)
**Age**	
14–17	25 (11.3)
18–19	41 (18.5)
20–29	84 (37.8)
30–39	37 (16.7)
40–49	24 (10.8)
>50	10 (4.5)
Unknown	1 (0.4)
*Total*	*222*
**Nationality**	
Italian	141 (63.5)
European, other	30 (13.5)
African	18 (8.1)
North American	5 (2.3)
Central-South American	26 (11.7)
Asian	2 (0.9)
*Total*	*222*

**Table 2:** Consumption of substances in relation to the time elapsed from rape, the age of the woman, the type of aggressor and the presence of injuries.

	Consumption Reported in the Medical Records				
	Alcohol (%)	Both Alcohol and Drugs (%)	Drugs (%)	No Intake (%)	No Data in the Report (%)
*Total (% out of 222)*	*86 (38.7)*	*33 (14.9)*	*36 (16.2)*	*4 (1.8)*	*63 (28.4)*
**Time elapsed**					
<6 h	3 (3.5)	0 (0.0)	3 (8.3)	3 (75.0)	8 (12.7)
6–12 h	36 (41.9)	12 (36.4)	3 (8.3)	0 (0.0)	12 (19.0)
12–24 h	19 (22.1)	7 (21.2)	9 (25.0)	0 (0.0)	8 (12.7)
24–48 h	12 (14)	2 (6.1)	5 (13.9)	0 (0.0)	4 (6.3)
>48 h	13 (15.1)	9 (27.3)	14 (38.9)	0 (0.0)	19 (30.2)
No data available	3 (3.5)	3 (9.1)	2 (5.6)	1 (25.0)	12 (19.0)
**Age range**					
14–17	2 (2.3)	0 (0.0)	8 (22.2)	0 (0.0)	14 (22.2)
18–19	19 (22.1)	11 (33.3)	5 (13.9)	1 (25.0)	5 (7.9)
20–29	45 (52.3)	8 (24.2)	11 (30.6)	1 (25.0)	19 (30.2)
30–39	12 (14.0)	11 (33.0)	4 (11.1)	1 (25.0)	10 (15.9)
40–49	8 (9.3)	3 (9.1)	4 (11.1)	1 (25.0)	8 (12.7)
>50	0 (0.0)	0 (0.0)	3 (8.3)	0 (0.0)	7 (11.1)
No data available	0 (0.0)	0 (0.0)	1 (2.8)	0 (0.0)	0 (0.0)
**Perpetrators**					
Unknown man	3 (3.5)	0 (0.0)	4 (10.3)	2 (50.0)	19 (30.2)
Partner	29 (33.7)	10 (30.3)	3 (7.7)	2 (50.0)	6 (9.5)
Known man	28 (32.6)	12 (36.4)	16 (41)	0 (0.0)	19 (30.2)
More assailants, known	2 (2.3)	5 (15.2)	5 (13.2)	0 (0.0)	1 (1.6)
More assailants, unknown	16 (18.6)	4 (12.1)	5 (13.2)	0 (0.0)	10 (15.9)
No data available	8 (9.3)	2 (6.1)	5 (13.2)	0 (0.0)	8 (12.7)
**Injuries**					
Injuries	83 (96.5)	32 (97.0)	16 (44.4)	2 (50.0)	23 (36.5)
No injuries	2 (2.3)	0 (0.0)	18 (50.0)	0 (0.0)	36 (57.1)
No data available	1 (1.2)	1 (3.0)	2 (5.6)	2 (50.0)	4 (6.3)

**Table 3:** Body areas reporting lesions, stratified by age group.

Age Range	14–17 n = 25 *(11.3)*	18–19 n = 41 *(18.4)*	20–29 n = 84 *(37.8)*	30–39 n = 37 *(16.7)*	40–49 n = 24 *(10.8)*	>50 n = 10 *(4.5)*	No Data Available n = 1 *(0.5)*	Total Lesions *(% out of 222)*
Head/neck	2	13	24	7	2	0	0	48 *(21.6)*
Trunk	0	1	2	1	1	1	0	6 *(2.7)*
Upper limbs	2	3	4	0	1	0	0	10 *(4.5)*
Lower limbs	1	1	3	4	1	0	0	10 *(4.5)*
Erogenous zones	1	4	6	1	1	1	0	14 *(6.3)*
Multiple area, but no erogenous zones	3	5	16	10	6	2	1	42 *(18.9)*
Multiple area and erogenous zones	1	7	10	3	3	1	0	25 *(11.3)*
No injuries	13	6	16	8	8	5	0	56 *(25.2)*
No data available	2	1	3	3	1	0	0	10 *(4.5)*

**Table 4:** Distribution of lesions in relation to the typology of the aggressor.

Typology of the Aggressor	Unknown Man n = 28 *(12.6)*	Partner n = 52 *(23.4)*	Known Man n = 75 *(33.8)*	More Aggressors, Known n = 13 *(5.9)*	More Aggressors, Unknown n = 34 *(15.3)*	No Data Available n = 20 *(9.0)*	Total Lesions *(% out of 222)*
Head/neck	1	22	16	3	4	2	48 *(21.6)*
Trunk	1	2	2	0	1	2	8 *(3.6)*
Upper limbs	1	2	2	0	6	1	12 *(5.4)*
Lower limbs	0	4	2	1	2	1	10 *(4.5)*
Erogenous regions	1	6	5	0	2	0	14 *(6.3)*
Multiple area, but no erogenous regions	4	10	16	2	8	3	43 *(19.4)*
Multiple area and erogenous regions	3	3	8	3	3	5	25 *(11.3)*
No injuries	15	3	22	4	6	6	56 *(25.2)*
No data available	2	4	2	0	2	0	10 *(4.5)*

**Table 5:** Results of the toxicological analysis.

Toxicological Results	Number of Women (%)
Negative results	81 (36.5)
Only alcohol	21 (9.5)
Only one drug	48 (21.6)
Multiple drugs	26 (11.7)
Alcohol and drugs	18 (8.1)
Alcohol and medications that do not affect the CNS	1 (0.4)
Drugs and medications that do not affect the CNS	27 (12.2)

**Table 6:** Distribution of substances revealed by toxicological analysis.

Toxicological Findings	Number of Positive Tests
Sedatives	48
Alcohol	40
Medicaments that do not affect the CNS	39
Cannabinoids	33
Anaesthetics	32
Antidepressants	15
Opioids	12
Antipsychotics	11
Other medicaments that affect the CNS	2

**Table 7:** Factors associated with a positive toxicological finding for alcohol at univariate and multivariable analysis.

Variable	Toxicological Analysis for Alcohol		Univariate Analysis	Multivariable Analysis
	Negative	Positive	OR (IC 95%)	OR (IC 95%)
Italian Yes No	59 (63.4%) 40 (87.0%)	34 (36.6%) 6 (13.0%)	**3.8 (1.4–10.3)**	0.3 (0.1–2.4)
Age class 14–19 years 20–39 years ≥40 years	22 (66.7%) 62 (73.8%) 15 (68.2%)	11 (33.3%) 22 (26.2%) 7 (31.8%)	0.7 (0.3–1.7) 0.9 (0.3–3.0)	-- --
Knowing her aggressor Yes No	62 (70.5%) 26 (72.2%)	26 (29.6%) 10 (27.8%)	1.1 (0.5–2.6)	--
Declared assumption of alcohol Yes No	44 (55.0%) 25 (96.2%)	36 (45.0%) 1 (3.9%)	**20.5 (2.2–187.4)**	**19.6 (1.5–259.1)**
Medical examination within 48 h Yes No	60 (62.5%) 30 (93.8%)	36 (37.5%) 2 (6.3%)	**9.0 (1.9–43.1)**	**6.1 (1.2–31.6)**
Amnesia Yes No	59 (64.8%) 30 (83.3%)	32 (35.2%) 6 (16.7%)	**2.7 (1.0–7.3)**	1.8 (0.6–6.0)
Physical lesions Yes No	70 (65.4%) 27 (90.0%)	37 (34.6%) 3 (10.0%)	**4.8 (1.3–17.4)**	2.1 (0.1–46.6)

## Data Availability

Not applicable.

## Supplementary Materials

The following supporting information can be downloaded at: https://www.mdpi.com/article/10.3390/ijerph192215090/s1, Table S1: Type of injuries detected; Table S2: Toxicological findings stratified by time from abuse to medical examination, age class, type of aggressor and presence of injuries; Table S3: number of women reporting different signs/symptoms at clinical examination; Item S1: raw data collection sheet; Item S2: details on psychic manifestations coding.
