# Supplementary material for: Sexual Harassments Related to Alcohol and Drugs Intake: The Experience of the Rape Centre of Turin
Source: Int J Environ Res Public Health. 2022 Nov 16;19(22):15090. doi: 10.3390/ijerph192215090 (PMC9690951; doi:10.3390/ijerph192215090)
Supplement: Supplementary file 1 [file ijerph-19-15090-s001.zip › ijerph-1990515-supplementary.pdf]

Supplementary materials

Sexual harassments related to alcohol and drugs intake: the experience of the Rape Centre of Turin

Mognetti et al.

Content list:

1. **Table S1**: Type of injuries detected
2. **Table S2**: Toxicological findings stratified by time from abuse to medical examination, age class, type of aggressor and presence of injuries.
3. **Table S3**: number of women reporting different signs/symptoms at clinical examination
4. **Supplementary Item S1:** raw data collection sheet
5. **Supplementary Item S2:** details on psychic manifestations coding

Table S1: Type of injuries detected.

**Type of injuries detected**	**Total n=117** (%)
Only blunt wounds	107 (91.5)
Only cold steel injuries	1 (0.8)
Blunt wounds and cold steel injuries	3 (2.6)
Blunt wounds and scars from previous episodes of gender violence	4 (3.4)
Only scars from previous episodes of gender violence	2 (1.7)

Note: In brackets, values are expressed as percentage of the total

Table S2: Toxicological findings stratified by time from abuse to medical examination, age class, type of aggressor and presence of injuries.

	Toxicological findings			
	**Alcohol**	**Both alcohol and drugs**	**Drugs**	**Negative results**
Total (% out of 222)	21 (9.5)	19 (8.6)	101 (45.5)	81 (36.5)
Time elapsed				
<6 h	2 (9.5)	2 (10.5)	8 (7.9)	5 (6.2)
6-12 h	15 (71.4)	11 (57.9)	19 (18.8)	18 (22.2)
12-24 h	2 (9.5)	2 (10.5)	23 (22.8)	16 (19.8)
24-48 h	1 (4.8)	1 (5.3)	11 (10.9)	10 (12.3)
>48h	0 (0.0)	2 (10.5)	30 (29.7)	22 (27.2)
No data available	1 (4.8)	1 (5.3)	10 (9.9)	10 (12.3)
Age range				
14-17	1 (4.8)	0 (0.0)	11 (10.9)	13 (16.0)
18-19	5 (23.8)	5 (26.3)	13 (12.9)	18 (22.2)
20-29	7 (33.3)	8 (42.1)	44 (43.6)	25 (30.9)
30-39	5 (23.8)	2 (10.5)	18 (17.8)	12 (14.8)
40-49	3 (14.3)	4 (21.1)	9 (8.9)	8 (9.9)
>50	0 (0.0)	0 (0.0)	6 (5.9)	4 (4.9)
No data available	0 (0.0)	0 (0.0)	0 (0.0)	1 (1.2)
Perpetrators				
Unknown man	1 (4.8)	1 (5.3)	14 (13.9)	12 (14.8)
Partner	6 (28.6)	3 (15.8)	27 (26.7)	16 (19.8)
Known man	5 (23.8)	10 (52.6)	29 (28.7)	30 (37.0)
More assailants, but known men	1 (4.8)	1 (5.3)	8 (7.9)	4 (4.9)
More assailants, but unknown men	4 (19.0)	4 (21.1)	12 (11.9)	14 (17.3)
No data available	4 (19.0)	0 (0.0)	11 (10.9)	5 (6.2)
Injuries				
Injuries	19 (90.5)	18 (94.7)	71 (70.3)	48 (59.3)
No injuries	2 (9.5)	1 (5.3)	28 (27.7)	25 (30.9)
No data available	0 (0.0)	0 (0.0)	2 (2.0)	8 (9.9)

Note: In brackets, values are expressed as percentage of the total

Table S3: number of women reporting different signs/symptoms at clinical examination

	Reported symptoms (n)
Multiples with amnesia	105
Nausea	83
Pains	47
Other	33
Amnesia	25
More physical symptoms	25
Multiples without amnesia	16
Confusion	6
Hallucinations	3
Vomiting / diarrhea	1
Dizziness	1
Weakness / exhaustion	0

4. **Supplementary Item S1:** raw data collection sheet

Page 1

Section	Fields
INFO GENERALI	codice paziente
	n. scheda clinica
	data evento
	luogo evento
	accesso SVS
	tempo intercorso
	note (dati salienti racconto)
ANAMNESI	nazionalità
	età
	terapie in corso
	tipologia farmaci
	posologia
ACCESSO PS	dati rilevanti
	terapie attuate
IGIENE	modalità
	cambio slip/vestiti
	minzione/ defecazione
	vomito
	pulizia cavo orale
RAPPORTI SESSUALI	Si/ no/quando
	data
EO GENERALE	sintomatologia
	altro

Page 2

Section	Fields
EO LESIONI	tipologia
	sede
	caratteristiche
	dimensione
	prognosi
EO GENITALE	lesioni
	note
ASSUNZIONE ALCOOL	volontaria
	obbligata
	inconsapevole
	tipologia
	quantità
ASSUNZIONE SOSTANZE	volontaria
	obbligata
	inconsapevole
	tipologia
	quantità
tossicologia NEGATIVA	matrice biologica
	intervallo
	sintomi/prelievo
	alcol
	benzodiazepine
	GHB
	sostanze

Page 3

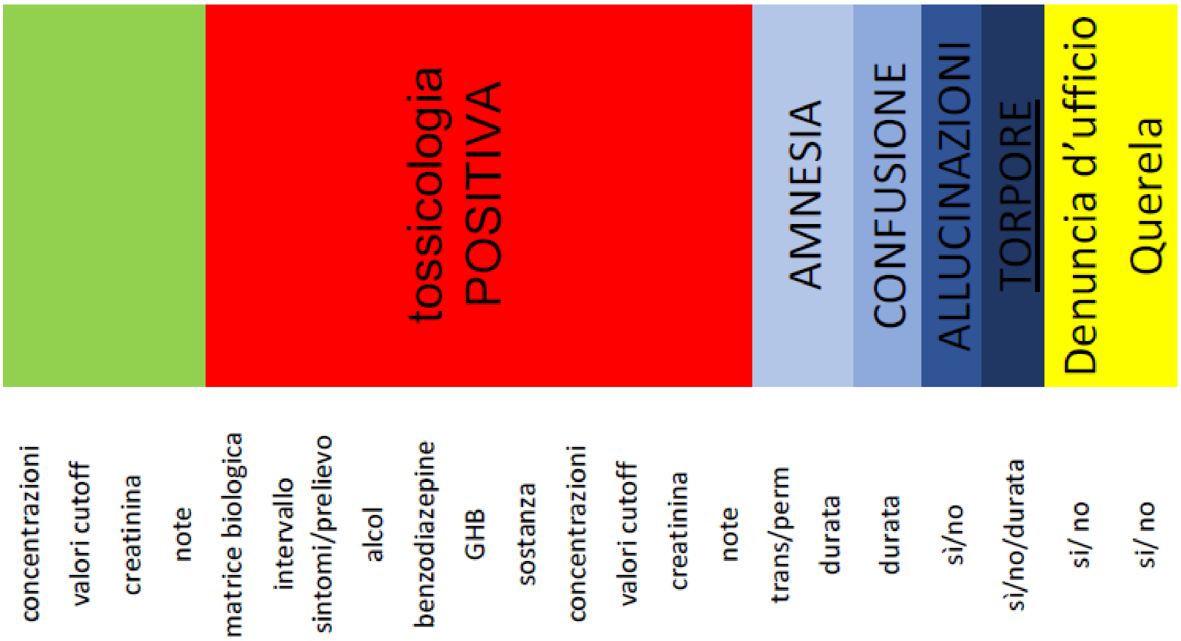

---

5. **Supplementary Item S2:** details on psychic manifestations coding

In the analysis phase, the recorded data on psychic manifestations were encoded as follows:

AMNESIA

yes/no

If yes Transitory=1
Permanent=2

If yes <6h=1
>6h=2

CONFUSION

yes/no

If yes <8h=1
>8h=2

HALLUCINATIONS

yes/no

NUMBNESS

yes/no

If yes <8h=1
>8h=2
